# Using Network Metrics in Soccer: A Macro-Analysis

**DOI:** 10.1515/hukin-2015-0013

**Published:** 2015-04-07

**Authors:** Filipe Manuel Clemente, Micael Santos Couceiro, Fernando Manuel Lourenço Martins, Rui Sousa Mendes

**Affiliations:** 1 Polytechnic Institute of Coimbra, Coimbra College of Education, Department of Education, Portugal.; 2 Faculty of Sport Sciences and Physical Education – University of Coimbra, Portugal.; 3 Ingeniarius, Lda., Coimbra, Portugal.; 4 Instituto de Telecomunicações (IT), Covilhã, Portugal.

**Keywords:** game analysis, soccer, network, metrics

## Abstract

The aim of this study was to propose a set of network methods to measure the specific properties of a team. These metrics were organised at macro-analysis levels. The interactions between teammates were collected and then processed following the analysis levels herein announced. Overall, 577 offensive plays were analysed from five matches. The network density showed an ambiguous relationship among the team, mainly during the 2nd half. The mean values of density for all matches were 0.48 in the 1st half, 0.32 in the 2nd half and 0.34 for the whole match. The heterogeneity coefficient for the overall matches rounded to 0.47 and it was also observed that this increased in all matches in the 2nd half. The centralisation values showed that there was no ‘star topology’. The results suggest that each node (i.e., each player) had nearly the same connectivity, mainly in the 1st half. Nevertheless, the values increased in the 2nd half, showing a decreasing participation of all players at the same level. Briefly, these metrics showed that it is possible to identify how players connect with each other and the kind and strength of the connections between them. In summary, it may be concluded that network metrics can be a powerful tool to help coaches understand team’s specific properties and support decision-making to improve the sports training process based on match analysis.

## Introduction

Different computer-based approaches have been attempting to extract and analyse tactical patterns in team sports (Grunz et al., 2012). Considering Memmert and Perl (2009), there are three ways of using a dynamically controlled network: i) as a static tool, if the application context does not change; ii) as an adaptive tool, if the application context changes and iii) as an object of analysis, if the learning dynamics of the network is of interest.

Many authors have suggested the use of the graph theory in sports (Duarte et al., 2012; Passos et al., 2011; Bourbousson et al., 2010). Bourbousson et al. (2010) used a graph theory to analyse the relationship between basketball players in each unit of attack crossing this quantitative analysis with a qualitative one to explain social interactions. Their main finding was the rise of a specific network regarding each team. These results suggest that the networks’ coordinations were built on local interactions that do not necessarily require all players to achieve the team’s goal. Despite the importance of social analysis, the network method can be used for performance analysis (Clemente et al., 2013). In spite of this, Duch et al. (2010) used the network approach to analyse passing accuracy, arc centrality and player performance during soccer matches.

The network application was also used for water polo analysis. Using the network method it was possible to identify the player who most frequently interacted with neighbouring teammates and the own contribution of the player to the successful and unsuccessful outcome of the collective performance (Passos et al., 2011). In their study, Passos et al. (2011) built an adjacency matrix for each attack unit, linking two levels: i) identification when a player passed the ball to a teammate, or ii) identification when players changed their positions due to a teammate’s displacement. The results suggest that network methods provide an interesting tool to qualitatively describe the interactions that occur between team players in the water polo game (Passos et al., 2011). Furthermore, it is possible to identify the preferential connections between players and their efficiency. Moreover, Duarte et al. (2012) suggested the definition of networks based on passing accuracy and positions’ switches.

Despite these achievements, many opportunities are still open in order to improve soccer analysis using the network approach. Actually, the network as a single analysis cannot provide a powerful quantitative analysis. Using the network analysis, however, does not allow one to identify the level of heterogeneity of the team or clusters inside the team. Many metrics should be suggested for further understanding the team’s behaviour. Therefore, this paper introduces a set of network metrics from the literature that can help in obtaining robust quantitative information about the team’s process. Using some approaches applied in other scientific fields (such as social sciences), a set of metrics is proposed to exploit the macro-analysis, characterising the homogeneity and distribution of the team’s global organisation.

It is expected to analyse some specific team properties such as the heterogeneity of players. Actually, the specific moments of the team during the match, as well as specific positions and roles of each player, decrease the possibilities to identify a high level of cooperation homogeneity between all teammates. To answer all these expectations, a set of metrics was applied on the same professional soccer team during five official matches.

## Material and Methods

### Sample

Five official matches of the same team of the First Portuguese Soccer League were analysed. The team won four matches and drew one. Overall, 21 players were analysed. Each player was encoded to identify the own characteristics, maintaining the same code for all matches (Table 1).

Despite different playing times for each player, this study aimed at keeping the real characteristics of an official soccer game, thus respecting the substitutions and the different options for each match. In order to overcome this task constraint associated with the specific dynamics of soccer, a network for each half of a match and for the whole match was performed, resulting in 15 different networks. This solution was considered so as to provide a useful and easy referential in a practical point of view. Actually, this option allowed us to consider that one player may not have played with another (due to substitutions). Nevertheless, this was a natural constraint from the real data collection. The same strategic distribution (1-4-2-3-1) was for all matches.

### Data Collection

An adjacency matrix was computed for each match. The adjacency matrix was used to build a finite *n* × *n* network where the entries represent the individual participation in the offensive play (*i.e.*, the network is developed considering the number of consecutive passes until the ball is lost). The offensive play considers all the passes from the same offensive sequence without losing ball possession. This option was based on Bourbousson et al. (2010) and Passos et al. (2011), who defined each ‘unit of attack’ (a soccer offensive play) starting at the moment a team gained ball possession until the ball was recovered by the opposing team. Overall, 577 offensive plays were analysed from five matches (Table 2).

The next section discusses the adjacency matrices obtained for each match and overall.

### Developing the Adjacency Matrix

A *MatLab* script denoted as *wgPlot* was developed by Wu (2009). Such a script allows one to plot graphs similarly to *gPlot*, a *MatLab* function that allows one to plot *n* nodes connected by links.

This represents a given *adjacency matrix*

$$A=\left[a_{ij}\right]\in {\mathbb{R}}^{n\times n}$$

defined by:

(1) $$a_{ij}=\{\begin{array}{ll} 1 & \mathrm{if}\;\mathrm{exist}\;\mathrm{connection}\;\mathrm{between}\;\mathrm{node}\;i\;\mathrm{and}\;j\\ 0 & \mathrm{if}\;\mathrm{no}\;\mathrm{exist}\;\mathrm{connection}\;\mathrm{between}\;\mathrm{node}\;i\;\mathrm{and}\;j \end{array}$$

It is noteworthy that in soccer, in which each adjacency matrix represents a successful pass, the diagonal elements (i.e., when *i* = *j*) are set equal to 1 to identify player *i* as one of the players that participated in the offensive play. As an example, consider the herein presented sequence of passes in which the first player corresponds to the first vertex and so on. The team under study has 11 players, *i.e.*, = 11 but the last five players did not contribute to this offensive play. The adjacency matrix of this offensive play would be represented by:

(2) $$A=\left[\begin{array}{ccccccccccc} 1 & 1 & 1 & 1 & 1 & 1 & 0 & 0 & 0 & 0 & 0\\ 1 & 1 & 1 & 1 & 1 & 1 & 0 & 0 & 0 & 0 & 0\\ 1 & 1 & 1 & 1 & 1 & 1 & 0 & 0 & 0 & 0 & 0\\ 1 & 1 & 1 & 1 & 1 & 1 & 0 & 0 & 0 & 0 & 0\\ 1 & 1 & 1 & 1 & 1 & 1 & 0 & 0 & 0 & 0 & 0\\ 1 & 1 & 1 & 1 & 1 & 1 & 0 & 0 & 0 & 0 & 0\\ 0 & 0 & 0 & 0 & 0 & 0 & 0 & 0 & 0 & 0 & 0\\ 0 & 0 & 0 & 0 & 0 & 0 & 0 & 0 & 0 & 0 & 0\\ 0 & 0 & 0 & 0 & 0 & 0 & 0 & 0 & 0 & 0 & 0\\ 0 & 0 & 0 & 0 & 0 & 0 & 0 & 0 & 0 & 0 & 0\\ 0 & 0 & 0 & 0 & 0 & 0 & 0 & 0 & 0 & 0 & 0 \end{array}\right]\in {\mathbb{R}}^{11\times 11}$$

The script *wgPlot* from Wu (2009) allows the user to input an adjacency matrix with weighted edges and/or weighted vertices being denoted as *edge-weighted edge-adjacency matrix A_w_*, introduced by Estrada (1995).

The weighted matrix *A_w_* can be easily defined by the sum of all adjacency graphs each one generated by a single offensive play. To allow a graphical representation of the players cooperation, the script presented by Wu (2009), denoted as *wgPlot*, was further extended based on the following features: *a*) the vertex (*i.e.*, player) size *i*, *i* = *j*, is proportional to the number of offensive plays player *i* participates in; *b*) the vertex (*i.e.*, cooperation between players) thickness *w_ij_* and *colormap* of the network is proportional to the number of offensive plays in which players *i* and *j*, *i* ≠ *j*, participate in together; *c*) the script receives as input a binary database (*e.g.*, excel file) in which each line corresponds to an offensive play and each column to a player, *i.e.*, each line corresponds to an adjacency matrix *A*; and *d*) besides returning the network from *A_w_*, it also returns the clusters, *i.e.*, sub communities, of the team based on Hespana’s work (Hespanha, 2004) and extensively used in Lim et al. (2005). This last point will be further explained in the next section.

### Seeking for Clusters within a Team

In order to detect groups among players, the graph theory has specific methodologies to constitute partitions. Uniform graph partition consists of dividing a graph into components, such that the components are of about the same size and there are few connections between the components. One of the functionalities of the graph partition is to generate communities (Couceiro et al., 2013). Communities, also called clusters or modules, are groups of vertices which probably share common properties and/or play similar roles within the graph (Fortunato, 2010).

The uniform graph partition has gained importance due to its application for clustering and detecting groups in social, pathological or biological networks (Fiduccia and Mattheyses, 1982). Commonly, graph partition is defined by *G* = (*V,E*) where *V* is the vertex and *E* is the edge, and partition *G* into smaller components with specific properties is possible. A *k*-partition of *V* is a collection *P* = {*V*_1_, *V*_2_,...,*V_k_*} of *k* disjoint subsets of *V*, whose union equals *V* (Hespanha, 2004).

The *MatLab* function *grPartition* described in the technical report of Hespana (2004) is able to perform fast partition of large graphs. This function implements a graph-partitioning algorithm based on spectral factorisation. The herein proposed *MatLab* script then merges the *wgPlot* and *grPartition* functions, with a few adaptations as previously presented, to understand players’ cooperation patterns within a given team.

Therefore, running the script with the previously described example would then return the following players network, thus identifying the players’ cooperation.

## Developing network metrics for soccer macro-analysis

Many kinds of networks (*e.g.*, biological, sociological or others) share some topological properties. To identify and describe such properties most potentially useful network concepts are known from graph theory (Couceiro et al., 2013). In soccer, one can divide network concepts into: *a*) intra-players network concepts (*i.e.*, network properties of a node); *b*) inter-player network concepts (*i.e.*, network relationships between two or more vertices); and *c*) group network concepts (*i.e.*, whole network concepts).

To allow the use of most of the network concepts, one can create a new relative weighted adjacency matrix *Ar* = [*r_ij_*]∈ R ^*n*×*n*^, defined as:

(3) $$r_{ij}=\{\begin{array}{ll} \frac{w_{ij}}{\text{max}\;A_{w}}, & \text{if}\;i\neq j\\ w_{ij}, & \text{if}\;i=j \end{array}$$

where 0 ≤ *r_ij_* ≤ 1 for *i* ≠ *j*, with *i,j* = 1,...,*n*. The denominator max*_i≠j_ A_w_* corresponds to the larger inter-player connectivity, *i.e.*, the players most participated together in the same offensive plays.

It is noteworthy that the diagonals of *A_r_* will still represent the number of offensive plays where a given player participated. However, this value is not considered to compute the network concepts herein presented.

For soccer analysis, one should first look at the ‘macro’ level. Therefore, the first step will be to understand how teams’ behave in a global way. Hence, after obtaining the adjacency matrix, three ‘macro’ metrics are proposed to identify the network density, heterogeneity and centralization.

### Network Density

The density measures the overall relationship among players. Therefore, more cooperative players yield a higher density.

To simplify the notation, let us use the function *vectorizeMatrix* (Horvath, 2011) which turns an *n* × *n* dimensional symmetric matrix *A* into a vector of which $\frac{n\left(n-1\right)}{2}$ components correspond to the upper-diagonal entries of *A* (Horvath, 2011):

(4) $$\mathrm{vectorizeMatrix}\left(A\right)=\left[a_{12}\;a_{22}\;\cdots a_{n-1,n}\right]$$

The network density is then defined as the mean of the off-diagonal entries of the adjacency matrix and is closely related to mean connectivity (a specific network measure) (Horvath, 2011). To that end, let us first define the connectivity of player *i* as:

(5) $$k_{i}=\sum_{i\neq j}r_{ij}$$

such that *k* = [*k*_1_ … *k_n_*] ∈ ℝ^1×*n*^ is the vector of the connectivity of players

The density can then be calculated as:

(6) $$\begin{array}{c} \mathrm{Density}\;=\;\mathrm{mean}\left(\mathrm{vectorizeMatrix}\left(A\right)\right)\\ =\frac{\Sigma_{t}\Sigma_{j>t}a_{tj}}{n\left(n-1\right)/2}\\ =\frac{\mathrm{mean}\left(k\right)}{n-1}\approx \frac{\mathrm{mean}\left(k\right)}{n} \end{array}$$

Considering soccer, values closer to 1 suggest that all players interact with each other, while a density of 0.5 indicates the presence of more ambiguous relationships.

### Network Heterogeneity

The *network heterogeneity* is closely related to the variation of connectivity across players (Albert et al., 2000; Watts, 2002). As in Horvath’s work (2011), this is defined as the coefficient of variation of the connectivity distribution, as follows:

(7) $$H=\sqrt{\frac{n\Sigma k_{t}^{2}-{\left(\Sigma k_{t}\right)}^{2}}{{\left(\Sigma k_{t}\right)}^{2}}},t=1,\ldots ,n$$

Many complex networks have been found to exhibit an approximate scale-free topology, which implies that these networks are highly heterogeneous. In other words, a high heterogeneity of a players’ network means that the soccer team exhibits a high level of sub communities and that there is, collectively, a low level of cooperation between players.

### Network Centralisation

*Network centralisation* measures the distribution level of a network. The individual centrality variation can predict the homogeneity level. If all players have the same centrality, the homogeneity level will be high. Network centralisation can be given by (Horvath, 2011).

Centralisation will be 0 for a network in which each node has the same connectivity and closer to 1 when players present a high heterogeneity. It should be highlighted that a regular grid network with *mean*(*k*) = *max*(*k*) has centralisation of 0.

(8) $$\begin{array}{l} \mathrm{Centralization}\;=\;\frac{n}{n-2}\left(\frac{\max(k)}{n-1}-\frac{\mathrm{mean}\left(k\right)}{n-1}\right)\\ =\frac{n}{n-2}\left(\frac{\max\left(k\right)}{n-1}-\mathrm{Density}\right)\\ \approx \frac{\max\left(k\right)}{n}-\mathrm{Density} \end{array}$$

For soccer analysis, centralization of 0 indicates that all players have the same level of interaction throughout the match. Nevertheless, a value closer to 1 suggests that one player can be the ‘master’ of the team, being the playmaker. Also, this means that the team has a partial tendency to play with this player, thus increasing heterogeneity and dependency on this player.

## Results

Table 3 depicts the frequency with which each player participated in the offensive plays for five matches. For better understanding of the results the strategic positions of each player can be seen in the following *sample section*.

In the overall time it is possible to observe that midfielders and lateral defenders participated in more offensive plays. On the other hand, despite the players being replaced throughout the match, forwards and strikers were the ones that participated least in the offensive plays. In the 1^st^ match, players 7 (48.1%), 2 (48.1%), 4 (45%) and 5 (42.7%) were the ones that participated the most in offensive plays, *i.e.*, respectively, the midfielder, right defender, central defender and left defender. In the 2^nd^ match, players 5 (36.8%), 6 (35.8%), 13 (35.8%) and 7 (34%) were the ones participating the most in offensive plays, *i.e.*, respectively, the left defender, defensive midfielder, left midfielder and midfielder. In the 3^rd^ match, players 6 (45.7%), 9 (40.5%), 2 (37.1%) and 19 (36.2%) were the ones presented in most of the offensive plays, *i.e.*, respectively, the defensive midfielder, right midfielder, right defender and left defender. In the 4^th^ match, it was the time for players 18 (42.7%), 14 (39.8%), 3 (38.8%) and 5 (38.8%) to show a higher participation in the offensive plays, *i.e.*, respectively, the central defender, midfielder, central defender and left defender. At last, in the 5^th^ match, players 4 (44.63%), 13 (38.84%) and 16 (37.19%) were the ones presenting larger offensive cooperation, respectively, the central defender, left midfielder and forward.

In all matches the number of players involved in offensive plays varied between 2 to 10 players. This can be observed by high values of the coefficients of variation that achieve levels of 53%. Besides the descriptive analysis, the network metrics that follow were also computed.

**Figure 1:**
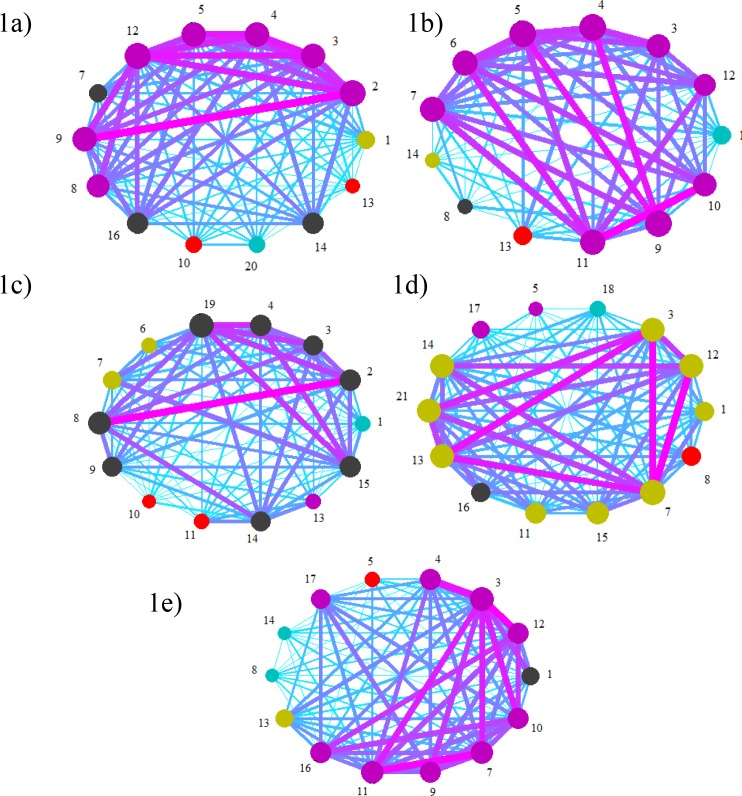
Network representation for all analysed matches (a: Overall Match 1; b: Overall Match 2; c: Overall Match 3; d: Overall Match 4; and e: Overall Match 5)

Therefore, based on the players involved in each offensive plays of the five analysed matches, 15 graphical networks representing the 1^st^ half, 2^nd^ half and the overall match were carried out (Figure 1). The size of the nodes (*i.e.*, players) represents the frequency of each player’s participation in offensive plays. Moreover, the size of the arrows represents the connectivity level between the nodes. The different node colours represent clusters that emerged from the team.

In the first match, the network graphics show higher connectivity between players 2 and 9 (right defender and right midfielder) and strong connectivity between all defensive players. Connectivity with the forward player (16) is not large, thus suggesting that the team was able to build its offensive plays based on defensive and midfield players. Also, the network density tends to decrease from the 1^st^ to the 2^nd^ half.

The network density seems to decrease in the 2^nd^ match. Moreover, the main connection between nodes is also different when compared to the 1^st^ match. In the 2^nd^ match larger connection can be found between the left midfielder (8) and the striker (11) in the 1^st^ half. Nevertheless, in the 2^nd^ half the larger connection is between players 7 (midfielder) and 9 (right midfielder). In this particular match, the relationship between teammates seemed to be less centralised and more distributed by all positions.

In the 3rd match, higher connectivity is between players 19 (left defender) and 4 (central defender) in the 1^st^ half, and players 8 (left midfielder) and 14 (midfielder) in the 2^nd^ half. Similarly with previous matches, the network density seems to be lower during the 2^nd^ half. Nevertheless, in this particular match the node’s sizes are much different, thus suggesting a different level of participation in offensive plays.

Different from all previous matches, the 4^th^ match shows a specific connection between players, suggesting a different team strategy. Larger connections in the 1^st^ half were between players 12 (right defender) and 16 (forward), 4 (central defender) and 16 (forward) and 5 (left defender) and 16 (forward). In the 2^nd^ half the connections between all players increased. Nevertheless, higher connections with the forward player may suggest a small change from previous matches. Actually, these results indicate that the team’s strategy was based on direct play and not on regular building play.

The last match analysed (5^th^ match) shows a higher connection between all players, mainly in the 1^st^ half. Nevertheless, these connections seem to have decreased during the 2^nd^ half, in which higher connectivity between players 12 (right defender) and 3 (central defender) can be observed. These results suggest a different strategy for the 2nd half, or just a lower opportunity to build regular offensive plays.

Although one could observe the connection between team players, a quantitative analysis was performed. Despite the regular network importance, further metrics need to be applied to have better understanding of team’s connectivity. Therefore, all the metrics introduced in the section 3 were explored.

### ‘Macro’ Analysis

The ‘macro’ analysis shows the results of the general connectivity level between all players (Table 5). Therefore, the aim of this analysis was to provide some quantitative information about the overall connection of players in the network and describe them between the first and second half.

The network density shows an ambiguous relationship among the team, mainly during the 2^nd^ half. Only in the 5th match did the value overcome 0.5, showing a higher interaction between all teammates. Nevertheless, the mean values for all matches were 0.48 in the 1^st^ half, 0.32 in the 2^nd^ half and 0.34 for the whole match. Those results suggest the existence of an ambiguous relationship between teammates to create offensive plays, possibly also suggesting some clusters in the team or some differences between the weights of each player during the offensive plays.

Therefore, network heterogeneity was used to assess the diversity of the players’ intervention. The heterogeneity coefficient for the overall matches equalled 0.47. Nevertheless, as previously suggested by the network density, the heterogeneity increased in all matches in the 2^nd^ half. In some cases, such as in the 1^st^, 4^th^ and 5^th^ matches, the coefficients increased until 84.74%. This heterogeneity suggests that offensive plays can be centralised in some players. Thus, network centralisation was performed.

The centralisation values show that there was no ‘star topology’. The results suggest that each node (i.e., each player) had nearly the same connectivity, mainly in the 1^st^ half. Nevertheless, the values increased in the 2^nd^ half, showing a decreasing participation of all players at the same level. Despite this tendency, it should be emphasised that only values closer to 1 mean a ‘star topology’. Thus, the higher value found (0.3182) in the 2^nd^ match indicated that the team did not depend on one single player to build their offensive plays.

## Discussion

A team can be characterised as a set of players that interact in a dynamic, interdependent and adaptive way working towards a common goal (Salas et al., 1992). One of the new measures proposed for analysing collective behaviour has been the network method (Bourbousson et al., 2010; Passos et al., 2011). Despite this important step forward, many applications could be developed to improve the network method’s potential. Therefore, this paper aimed to analyse the network properties by applying some metrics.

A set of metrics based on the graph theory could be applied. Nevertheless, only three were used to classify the football team’s structure. By using the density network it was possible to measure how the players interacted with each other. Despite a small number of publications regarding the network metrics applied to soccer, Grund (2012) studied the correlation between two network properties (density and centralisation) and a team’s performance based on goals scored in 760 matches in the English Premier League. The main results showed that high levels of interaction between teammates (density) led to increased team performance. Moreover, centralised interaction patterns led to decreased team performance. In fact, even in a social context, teams with denser networks had a tendency to perform better and remain more viable (Balkundi and Harrison, 2006).

In our study an overall network computation during five consecutive matches was analysed. The overall results suggested a moderate level of density in the 1^st^ half, and a lower value in the 2^nd^ half. Actually, in all matches the density level decreased, thus two main explanations can be addressed. The first explanation can be related with the team’s strategy alteration, decreasing the participation of all players in offensive plays, trying to avoid accumulating fatigue. By decreasing the number of players involved it was possible to allow some players to rest actively. Nevertheless, other explanations can also be addressed. The frequency of the direct play was increased in the 2^nd^ half. Thus, due to the increase in direct play, more clusters emerged because the offensive plays involved many players. The building play can on the other hand explain the higher density values shown in the 1^st^ half which involved more players. Thus, the types of offensive plays can explain the connection density. The direct play may have increased centralisation among some players. The direct play involved a lot of participation from forwards and strikers. To study the individual contribution of players the network centralisation metric was performed.

Generally in a social context the centralised network systems are found to be negatively associated with team performance (Cummings and Cross, 2003). The centralisation values obtained from the five matches do not suggest high centralisation. Despite the increasing tendency towards that in the 2^nd^ half, the values are too small to consider high centralisation. This can be important in order to understand that the analysed team had more options and did not depend only on a specific player; thus it was not a star graph (Wasserman and Faust, 1994). Nevertheless, the notion of dependency and prominence is always present in team sports. Taking such ideas into consideration, the centralisation levels in particular players were studied by Malta and Travassos (2014). In their experimental approach only the attacking transition situations (defence-attack) were analysed and then they processed the individual contributions of each player for the general network of the team. For such analysis they used the in-degree and out-degree centrality metrics that measure the total number of passes received and performed during the match. The results were particularly interesting because of the possibility to find the most prominent players for the attacking process. When a supported play style was concerned, it was found that the defensive midfielder received more balls from the teammates, therefore with regard to a direct play style, the centre forward received more balls from the teammates. Briefly, these results reveal that centralisation may depend on a kind of the play style adopted by the team (Clemente et al., 2014).

Besides the density and centralisation values, also the heterogeneity levels of the team during five matches were studied. High heterogeneity of the players’ network means that the soccer team exhibits a high level of sub communities, i.e., clusters.

Some studies have used the clustering coefficient to classify the network properties of soccer teams (Peña and Touchette, 2012; Cotta et al., 2013). In the study of Peña and Touchette (2012) two specific matches were analysed during FIFA World Cup 2010: Spain vs. the Netherlands and Germany vs. Uruguay. With regard to the match of Spain and the Netherlands, similar levels of clustering coefficients were found; nevertheless, the Spanish team had the highest levels of clustering coefficients in midfielders and in the Netherlands the highest values were found in the goalkeeper and defender. With respect to the match of Germany vs. Uruguay, the German team had the highest values of clustering coefficients. In our study it was not clustering that was analysed but heterogeneity. The results showed an increasing tendency for clustering in the 2^nd^ half in all analysed matches. Thus, this value means that the offensive plays did not involve all players at the same level. This is actually understandable considering the specific roles of the players. The offensive plays can ‘emerge’ in many specific areas and these areas determine the interaction between teammates. If the ball possession is obtained in the defensive zone it is more possible that the offensive play will involve more players. On the other hand, if the ball is recovered closer to the opponent’s goal, a higher involvement of all players is less predictable. Thus, defensive pressing and the team’s attacking style will determine their heterogeneity and density, as well as centralisation. In fact, regarding the clustering coefficient analysis performed on the basis of the FIFA World Cup 2010 final it was found that the clustering coefficient was much lower (with more clusters) in the 2^nd^ part of extra time (Cotta et al., 2013). The authors suggested that such an event can be justified by the less structured development of the game and larger distances between team lines.

Another important variable to be discussed along with these ‘macro’ analysis parameters can be the score. In all five matches the analysed team drew one and won the remaining matches. Nevertheless, for many teams the score can be also important in order to improve the team’s strategy. Perhaps the more direct play in the 2^nd^ half analysed using the network method may indicate a strategy to achieve the goal. On the other hand, if the team has a positive score from the 1^st^ match, one of their options can be to choose a more conservative approach such as involving more counter-attacks in order to reduce the level of connectivity between all players and increase heterogeneity and centralisation. In fact the dynamics of the game can lead to a deep change in the applied pattern of play (Gréhaigne et al., 1997), thus constraining the style of play and then the network properties.

Only general properties of a network were analysed in this study. For future research it is important to add the individual centrality measures to complement the general properties of a graph. Such an analysis will help identify how players contribute to the general cooperation levels of the team. Moreover, some performance variables such as match status, possession of the ball or goals scored (Lago-Peñas and Dellal, 2010) can be used to associate with the network properties, thus providing better global understanding that explains the obtained results.

## Conclusion

In this work, the authors proposed a set of network metrics to improve the offensive processes analysis of soccer teams. Five official matches were analysed from the same team during their national championship competition. The density, heterogeneity and centralisation metrics were used for a ‘macro’ analysis. These metrics showed that it is possible to identify how players connect with each other and the kind and strength of the connections between them. All the metrics applied showed a high level of importance towards the identification and characterisation of the team’s collective process. Moreover, the network approach proposed in this paper showed its quick and easy application that can provide useful information for coaches, supporting their decisions about the soccer training process. By describing and discussing the interpretation of each network an easy and powerful tool can be acquired in order to enhance the knowledge about the team’s behaviour. This can be used in an online or offline fashion. In sum, the network approach based on the graph theory provides a useful method for measuring and analysing the communication between teammates, thus helping to understand the team’s processes and own properties.

## Figures and Tables

**Table 1:** Description of the analysed players and their time per match

	Position	1^st^ Match	2^nd^ Match	3^rd^ Match	4^th^ Match	5^th^ Match	Overall
Player 1	Goalkeeper	90	90	90	90	90	450
Player 2	Right Defender	90	0	90	0	0	180
Player 3	Central Defender	90	90	90	90	90	450
Player 4	Central Defender	90	90	90	0	90	360
Player 5	Left Defender	90	90	0	90	90	360
Player 6	Defensive Midfielder	0	90	90	0	0	180
Player 7	Midfielder	90	90	45	28	27	280
Player 8	Left Midfielder	60	90	71	8	74	303
Player 9	Right Midfielder	90	38	90	0	27	245
Player 10	Forward	90	26	90	0	16	222
Player 11	Striker	0	64	63	45	63	235
Player 12	Right Defender	67	90	0	90	90	337
Player 13	Left Midfielder	23	90	27	90	90	320
Player 14	Midfielder	23	52	45	82	63	265
Player 15	Midfielder	0	0	19	45	0	64
Player 16	Forward	67	0	0	90	90	247
Player 17	Defensive Midfielder	0	0	0	90	90	180
Player 18	Central Defender	0	1	0	90	0	91
Player 19	Left Defender	0	0	90	0	0	90
Player 20	Striker	30	0	0	0	0	30
Player 21	Right Midfielder	0	0	0	62	0	62

**Table 2:** Frequency of offensive plays in each match

	1^st^ Match	2^nd^ Match	3^rd^ Match	4^th^ Match	5^th^ Match	Overall
1^st^ Half	58	65	56	49	65	293
2^nd^ Half	73	41	60	54	56	284
Overall	131	106	116	103	121	577

**Table 3:** The number of participations of each player per offensive plays

	Position	1st Match			2nd Match			3rd Match			4th Match			5th Match		
		1^st^ H	2^nd^ H	Total	1^st^ H	2^nd^ H	Total	1^st^ H	2^nd^ H	Total	1^st^ H	2^nd^ H	Total	1^st^ H	2^nd^ H	Total
Player 1	Goalkeeper	9	12	21	7	8	15	10	10	20	11	11	22	13	13	26
Player 2	Right Defender	34	29	63	0	0	0	22	21	43	0	0	0	0	0	0
Player 3	Central Defender	23	30	53	17	9	26	12	22	34	18	22	40	21	20	41
Player 4	Central Defender	29	30	59	21	10	31	24	17	41	0	0	0	27	27	54
Player 5	Left Defender	28	28	56	25	14	39	0	0	0	20	20	40	21	15	36
Player 6	Defensive Midfielder	0	0	0	26	12	38	29	24	53	0	0	0	0	0	0
Player 7	Midfielder	31	32	63	25	11	36	19	0	19	0	11	11	0	11	11
Player 8	Left Midfielder	20	7	27	17	18	35	23	8	31	0	6	6	20	9	29
Player 9	Right Midfielder	29	26	55	0	9	9	19	28	47	0	0	0	0	8	8
Player 10	Forward	21	22	43	0	9	9	18	16	34	0	0	0	0	6	6
Player 11	Striker	0	0	0	13	3	16	7	6	13	13	0	13	15	6	21
Player 12	Right Defender	28	12	40	25	11	36	0	0	0	15	22	37	25	14	39
Player 13	Left Midfielder	0	18	18	23	15	38	0	17	17	14	24	38	24	23	47
Player 14	Midfielder	0	20	20	28	3	31	0	36	36	20	21	41	23	13	36
Player 15	Midfielder	0	0	0	0	0	0	0	19	19	0	23	23	0	0	0
Player 16	Forward	22	13	35	0	0	0	0	0	0	11	14	25	23	22	45
Player 17	Defensive Midfielder	0	0	0	0	0	0	0	0	0	15	15	30	26	13	39
Player 18	Central Defender	0	0	0	0	0	0	0	0	0	20	24	44	0	0	0
Player 19	Left Defender	0	0	0	0	0	0	25	17	42	0	0	0	0	0	0
Player 20	Striker	0	11	11	0	0	0	0	0	0	0	0	0	0	0	0
Player 21	Right Midfielder	0	0	0	0	0	0	0	0	0	16	6	22	0	0	0

**Table 4:** Descriptive statistics of the number of players involved in the offensive plays

	1st Match			2nd Match			3rd Match			4th Match			5th Match		
	1^st^ H	2^nd^ H	Total	1^st^ H	2^nd^ H	Total	1^st^ H	2^nd^ H	Total	1^st^ H	2^nd^ H	Total	1^st^ H	2^nd^ H	Total
Median	4.00	3.00	4.00	3.00	3.00	3.00	3.00	3.50	3.00	3.00	3.00	3.00	3.00	3.00	3.00
Mean	4.72	3.97	4.31	3.49	3.22	3.39	3.71	4.02	3.87	3.53	4.06	3.81	3.66	3.57	3.62
Standard Deviation	2.19	1.97	2.10	1.86	1.62	1.77	1.68	2.07	1.89	1.56	2.34	2.01	1.78	1.37	1.60
Coefficient of Variation	0.46	0.50	0.49	0.53	0.50	0.52	0.45	0.52	0.49	0.44	0.58	0.53	0.49	0.38	0.44

**Table 5:** Macro’ analysis results for all 5 matches

Matches	Network Density			Network Heterogeneity			Network Centralization		
	1^st^	2^nd^	Overall	1^st^ Half	2^nd^ Half	Overall	1^st^ Half	2^nd^ Half	Overall
1^st^ Match	0.5115	0.3302	0.3432	0.2633	0.4551	0.4880	0.1773	0.2476	0.2439
2^nd^ Match	0.3970	0.2835	0.3795	0.3429	0.3900	0.4513	0.1599	0.2012	0.2114
3^rd^ Mach	0.4364	0.3141	0.3221	0.3808	0.4290	0.4182	0.2652	0.3182	0.2277
4^th^ Mach	0.4579	0.4103	0.3199	0.2562	0.4213	0.4834	0.1848	0.2965	0.2687
5^th^ Match	0.5846	0.2612	0.3473	0.2419	0.4469	0.4901	0.2162	0.2821	0.3127
Overall mean	0.4775	0.3199	0.3424	0.2970	0.4285	0.4662	0.2007	0.2691	0.2529
